# Chronic nonbacterial osteomyelitis in children

**DOI:** 10.1186/1546-0096-9-S1-P23

**Published:** 2011-09-14

**Authors:** A Desdoits, S Gandon-Laloum

**Affiliations:** 1 Service de Chirurgie Pédiatrique, Caen, France

## 

Chronic Nonbacterial Osteomyelitis (CNO) is an autoinflammatory disease that presents as recurrent flares of bone pain, sometimes febrile and with lytic radiological pictures. Extraarticular manifestations are also possible. We present a retrospective study of eleven children (ten females, one male), followed in Pediatric Orthopedic ward of the Caen University Hospital between 2001 and 2011. We report the initial symptoms, the biological profiles and the radiological findings which led to the diagnosis of CNO. Secondly, we describe treatment and disease’s evolution of these patients.

The mean age at CNO onset in our study was 10.5 years (4 to 14). Girls were affected more than boys (10/1). The mean term to the diagnosis was ten months (one week to five years). The most suggestive feature was the involvement of the metaphyses of the long bone (tibia and femur), but all bones could be involved (table 1). Eight patients underwent a bone biopsy. Eleven patients had a radionuclide bone scanning, with asymptomatic lesions detected.

**Table 1:** bone involvement at the CNO onset and at the end of follow-up

	Diagnosis	End of follow-up
n=	11	11

Anterior chest wall	6	8
-Clavicle	3	5
-Rib	2	2
-Sternum	1	1
Vertebrae	5	8
Pelvis	5	6
Femur	6	6
Tibia	7	9
Calcaneus	4	4
Mandible	1	1
Radius	1	1
Humerus	1	1
Skull	1	2

At the end of the follow-up, the involvement of the spine is more frequent than at diagnosis (table1). Five patients had an articular involvement, before the CNO onset, or later in the evolution. One patient had a palmoplantar pustulosis, and one a psoriasis.

Only six patients had an increased C-reactive protein level, and seven an increased erythrocyte sedimentation rate at the CNO onset. All are treated by NSAIDs: seven children had regular attacks of pain, four were in remission. Two patients had an inequal length of inferior limbs, and two patients had vertebral deformities.

In conclusion, the patients of our study had the same characteristics as patients in the literature. A better comprehension of the physiopathology seems to classify CNO as auto-inflammatory diseases, even though some patients evolve into spondylarthropathy.

